# Supplementary material for: A high-resolution mRNA expression time course of embryonic development in zebrafish
Source: eLife. 2017 Nov 16;6:e30860. doi: 10.7554/eLife.30860 (PMC5690287; doi:10.7554/eLife.30860)
Supplement: Supplementary file 6. [file elife-30860-supp6.zip › biolayout-clusters-files/Cluster225-genes.html]

Cluster225


# Cluster225: Genes

| Ensembl ID | Gene Name | Chr | Start | End | Biotype |
| --- | --- | --- | --- | --- | --- |
| ENSDARG00000020761 | arrdc2 | 11 | 13466845 | 13476123 | protein\_coding |
| ENSDARG00000016483 | baiap2l2a | 3 | 24306532 | 24327733 | protein\_coding |
| ENSDARG00000013858 | cdc14aa | 2 | 7203614 | 7233926 | protein\_coding |
| ENSDARG00000016415 | dhtkd1 | 25 | 7907907 | 7931137 | protein\_coding |
| ENSDARG00000036588 | mhc1zba | 1 | 46464192 | 46470764 | protein\_coding |
| ENSDARG00000035949 | tmem106bb | 19 | 31805037 | 31815116 | protein\_coding |
